# Diffusion Tensor Imaging Tractography of White Matter Tracts in the Equine Brain

**DOI:** 10.3389/fvets.2020.00382

**Published:** 2020-07-30

**Authors:** Samuel Boucher, Germain Arribarat, Benjamin Cartiaux, Elodie Anne Lallemand, Patrice Péran, Alexandra Deviers, Giovanni Mogicato

**Affiliations:** ^1^ToNIC, Toulouse NeuroImaging Center, Université de Toulouse, INSERM, UPS, Toulouse, France; ^2^INSERM UMR1037, Cancer Research Center of Toulouse, Oncopole, Toulouse, France; ^3^INTHERES, Université de Toulouse, INRA, ENVT, Toulouse, France; ^4^ToNIC, Toulouse NeuroImaging Center, Université de Toulouse, INSERM, UPS, ENVT, Toulouse, France

**Keywords:** MRI, DTI, tractography, equine, white matter tracts

## Abstract

Tractography, a noninvasive technique tracing brain pathways from diffusion tensor magnetic resonance imaging (DTI) data, is increasingly being used for brain investigation of domestic mammals. In the equine species, such a technique could be useful to improve our knowledge about structural connectivity or to assess structural changes of white matter tracts potentially associated with neurodegenerative diseases. The goals of the present study were to establish the feasibility of DTI tractography in the equine brain and to provide a morphologic description of the most representative tracts in this species. *Postmortem* DTI and susceptibility-weighted imaging (SWI) of an equine brain were acquired with a 3-T system using a head coil. Association, commissural, and projection fibers, the three fiber groups typically investigated in tractography studies, were successfully reconstructed and overlaid on SWI or fractional anisotropy maps. The fibers derived from DTI correlate well with their description in anatomical textbooks. Our results demonstrate the feasibility of using *postmortem* DTI data to reconstruct the main white matter tracts of the equine brain. Further DTI acquisitions and corresponding dissections of equine brains will be necessary to validate these findings and create an equine stereotaxic white matter atlas that could be used in future neuroimaging research.

## Introduction

Diffusion tensor imaging (DTI) is one of the most popular and widely used (1) MRI techniques in brain research to describe the orientation of white matter fibers. The process of fiber tracking, called tractography, allows for a virtual dissection and three-dimensional representation of white matter tracts (2).

As DTI tractography assesses the structural integrity of white matter, it has been widely used for prognostic and/or diagnostic purposes in various brain pathologies such as stroke (3–6), neurodegenerative diseases (7–11), and brain tumors (12). With the increasing availability of high-field-strength MRI (1.5 and 3 T) in veterinary facilities (13–17), the use of this technique is now gradually growing for the description of white matter anatomy and structural connectivity of domestic mammals (dog, cat, ferret, and sheep) (18–27). These collected anatomical data are of particular interest as they might have applications in both veterinary medicine and experimental research. Indeed, large animals are now increasingly seen as valuable models with regard to comparative neuropathology because they can spontaneously reproduce some human brain diseases [such as Alzheimer's disease (28, 29), Parkinson's disease (30, 31), lysosomal storage diseases (32, 33), or gliomas (34)] and be examined with the same MRI devices used in human medicine.

However, the validation of tractography in large animals requires prior knowledge of the morphology of white matter tracts. In a recent study, Pascalau et al. (35) used a fiber dissection technique to provide a detailed anatomical description of the main association, commissural, and projection fibers of the dog, cat, and horse. This study, which was the first to describe the spatial anatomy of white matter tracts in the horse, has still not been complemented by DTI tractography in the equine brain. Yet poorly understood neurodegenerative diseases affecting horses such as equine degenerative myeloencephalopathy or nigropallidal encephalomalacia would greatly benefit from the use of DTI tractography as this technique should identify damages in specific white matter tracts and correlate them with clinical symptoms. DTI tractography could also be used in healthy brains in order to improve our knowledge about anatomy and function of the equine brain by mapping its structural connectivity.

Tractography, the only noninvasive way to capture the three-dimensional anatomy of white matter tracts, appears as an exciting method to study the equine brain in the clinical or research setting. In this context, the goal of the present study was to evaluate the ability of DTI tractography to characterize the equine brain fiber bundles. To achieve this, the reconstruction of the most representative tracts was investigated, and the fiber tracking results were compared to the description of these tracts in the literature.

## Methods

### Animal Sampling

A 12-year-old French Standardbred mare (bodyweight = 553 kg) was euthanized for medical reasons unrelated to neurological disease. Immediately after euthanasia, the brain was extracted from the skull and fixed for 1 month in 10% formalin solution.

### MRI Acquisition and Preprocessing

MRI examination was performed at the Institute for Brain Sciences of Toulouse using a high-field 3.0-T magnet (Philips ACHIEVA dStream) and a head coil for signal reception. Twenty-four hours before MRI acquisition, the brain was rinsed with water and submerged in a 0.9% saline solution (NaCl). Just before starting acquisition, the brain was placed in an MRI-compatible container (zip-locked hermetic plastic bag) totally filled with saline solution. After being gently agitated in order to manually remove air bubbles, the bag was closed and then placed in a foam mold. The space between the bag and the concavity of the mold was filled with cotton balls in order to prevent motion of the bag during acquisition (36). The imaging protocol comprised T1-weighted images using a gradient-echo sequence (repetition time 7.72 ms; echo time 3.64 ms; voxel size 1 × 1 × 1 mm, and matrix 240 × 240 × 180); T2-weighted images using a spin-echo sequence (repetition time 286 ms; echo time 1,500 ms; voxel size 1 × 1 × 1 mm, and matrix 240 × 240 × 180); susceptibility-weighted imaging (SWI) using a gradient-echo sequence [repetition time 186 ms; multi-echo (*n* = 22; TE1 = 3.20; dTE = 2.70 ms), voxel size 0.7 × 0.7 × 1 mm, and matrix 288 × 288 × 200); and diffusion-weighted images using a spin-echo sequence (repetition time 11.5 s, echo time 76.1 ms, flip angle 90°, voxel size 1.97 × 1.97 × 2 mm, matrix 112 × 112 × 48, 64 independent directions, and *b*-value 3,000 s/mm^2^). The diffusion sequences were implemented from preexisting sequences that had already been used in the laboratory to carry out tractography of an *ex vivo* equine brain (37). The acquisition time was 12 min, which was repeated five times for averaging, for a total acquisition time of 1 h. Raw diffusion-weighted data were denoised using an LPCA filter with a Rician noise model (38) on MATLAB (MathWorks, Inc., MA, USA). The data were then corrected for geometric distortion due to eddy currents using DSI Studio. T1- and T2-weighted images were registered to b0 images using the C++ toolbox ANTS (39). T1-weighted, T2-weighted, and susceptibility-weighted images were useful for anatomical reference and three-dimensional rendering of the brain. T2-weighted images were registered to b0, and then T1-weighted images were registered to T2-weighted images. The susceptibility-weighted images were first down-sampled to match the resolution of the DTI images, and then a linear rigid-body registration based on the ANTS registration algorithm was performed (39). These “down-sampled” susceptibility-weighted images, which were perfectly realigned with the DTI, were used to guide region-of-interest (ROI) placement. On the other hand, we decided to use the original high-resolution susceptibility-weighted images for the presentation of ROI and tracking results, due to the excellent visual quality of these images. We used the linear affine registration of DSI Studio to realign the high-resolution SWI with the DTI, through the down-sampled SWI. Even if the high-resolution SWI was not perfectly realigned, these images allow a better visualization of the results.

### DTI Reconstruction

For this study, the reconstruction was done on DSI Studio; the DTI reconstruction follows the Basser method (40). To model the diffusion phenomenon, the diffusion tensor model described by Basser et al. (41) was used. This model is based upon a three-dimensional model of Gaussian diffusion displacements.

$$\begin{array}{l} D=\left(\begin{array}{ccc} D_{xx} & D_{xy} & D_{xz}\\ D_{yx} & D_{yy} & D_{yz}\\ D_{zx} & D_{zy} & D_{zz} \end{array}\right) \end{array}$$

*D* is calculated for each voxel based on the b0 reference image and all the diffusion-weighted images. To obtain a visualization of the tensor, the matrix *D* needs to be diagonalized to obtain the three eigenvalues λ_1_ λ_2_ λ_3_ and the three eigenvectors v_1_ v_2_ v_3_. So diffusion tensor can be described as an ellipsoid.

### Mean Diffusivity and Fractional Anisotropy

Mean diffusivity (MD) consists in the calculation of the mean value of the three eigenvalues. This leads to a parametric map of the diffusivity for each voxel, without taking into account the direction of diffusion. Once MD has been calculated, fractional anisotropy (FA) can be retrieved. FA comprised between 0 and 1, 0 being a voxel where the diffusion is totally isotropic and 1 an anisotropic voxel (one direction is preponderant).

$$\begin{array}{l} FA=\sqrt{\frac{3}{2}}\;\sqrt{\frac{{\left(\lambda_{1\;}-MD\right)}^{2}+{\left(\lambda_{2}-MD\right)}^{2}+{\left(\lambda_{3\;}-MD\right)}^{2}}{{\lambda_{1\;}}^{2}+{\lambda_{2}}^{2}+{\lambda_{3\;}}^{2}}} \end{array}$$

Since water diffusion is restricted in white matter tracts, a voxel containing fibers will have a high FA value. The three values of the first eigenvector v_1_ were assigned to the red, green, and blue channel to obtain a colored image for which every single color represents a distinct fiber orientation (red: right–left, green: ventral–dorsal, and blue: rostral–caudal).

### Tractography

A deterministic tracking method was chosen (42) as it seems to achieve the highest valid connection compared to other fiber tracking approaches. As only one brain was scanned in our study, we assume that a deterministic tracking should improve stability and be less subjected to individual differences than a probabilistic tracking.

### ROI and Region-of-Avoidance (ROA) Delineation

For each tract, ROIs were delineated manually, and sometimes one or several ROAs were additionally placed in order to specifically segregate fibers of interest. Regions were placed using anatomical descriptions of T1- and T2-weighted images of the equine brain (14, 15), dissection of major white matter tracts in the equine brain (35), and a human tractography atlas (2). Color-coded FA maps and down-sampled SWI were used to place ROI and ROA. Sometimes, ROIs were used as seed for better results. ROIs were delineated on dorsal planes for the arcuate fasciculus, the uncinate fasciculus, the inferior longitudinal fasciculus, and the inferior fronto-occipital fasciculus; on transversal planes for the cingulum and internal capsule; and on both sagittal and transversal planes for commissural fibers. Representative color-coded FA maps and three-dimensional representation of ROIs are displayed in Supplementary Figures 1, 2.

## Results

Association, commissural, and projection fibers are the three fiber groups typically investigated in tractography studies. White matter tracts of these three groups were successfully reconstructed in our study.

### Association Fibers

**Figure 1:**
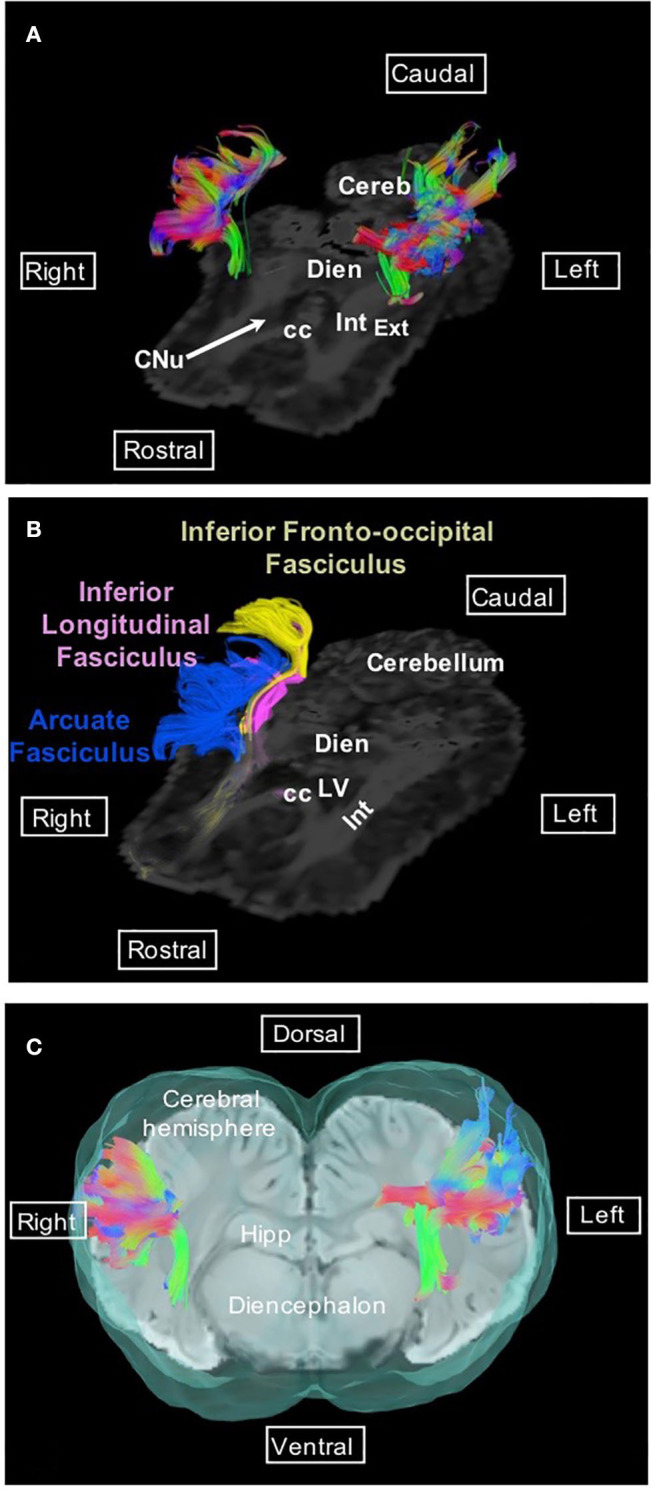
Arcuate fasciculus. **(A)** Dorsal view of the arcuate fasciculus overlaid on the FA map. **(B)** Dorsal view of the arcuate fasciculus (blue), inferior longitudinal fasciculus (pink), and inferior fronto-occipital fasciculus (yellow) overlaid on the FA map. **(C)** Transversal view of the arcuate fasciculus overlaid on SWI and the isosurface of the brain. Directional colors are green, ventral to dorsal (or dorsal to ventral); blue, rostral to caudal (or caudal to rostral); red, right to left (or left to right). cc, corpus callosum; Cereb, cerebellum; CNu, caudate nucleus; Dien, diencephalon; Ext, external capsule; Hipp, hippocampus; Int, internal capsule; LV, lateral ventricle.

Association fibers, which connect distant or neighboring gyri in the same hemisphere, form different bundles; the most investigated in tractography studies are the superior and inferior longitudinal fasciculi, the uncinate fasciculus, the inferior fronto-occipital fasciculus, and the cingulum. The superior longitudinal fasciculus (or arcuate fasciculus) is a lateral bundle bending dorsally to the claustrum; it is composed of long and short fibers connecting Sylvian gyri to the occipital cortex (Figure 1).

**Figure 2:**
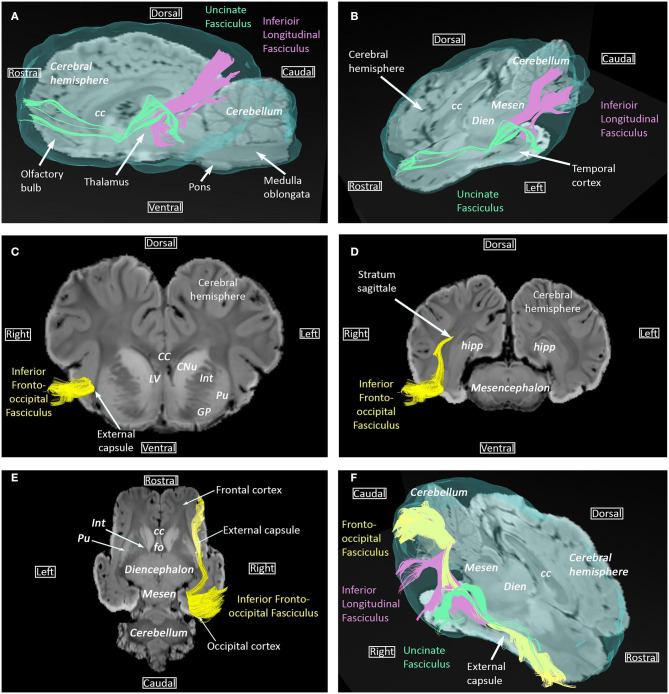
Inferior longitudinal fasciculus, uncinate fasciculus, and inferior fronto-occipital fasciculus. **(A)** Sagittal view of the uncinate (green) and inferior longitudinal (pink) fasciculi overlaid on SWI and the isosurface of the brain. **(B)** Dorsal and sagittal views of the uncinate (green) and inferior longitudinal (pink) fasciculi overlaid on SWI and the isosurface of the brain. **(C,D)** Transversal views of the inferior fronto-occipital fasciculus overlaid on SWI, at telencephalon and mesencephalon levels, respectively. **(E)** Dorsal view of the inferior fronto-occipital fasciculus overlaid on SWI. **(F)** Dorsal and sagittal views of the uncinate (green), inferior longitudinal (pink), and inferior fronto-occipital (yellow) fasciculi overlaid on SWI and the isosurface of the brain. cc, corpus callosum; CNu, caudate nucleus; Dien, diencephalon; fo, fornix; GP, globus pallidus; Hipp, hippocampus; Int, internal capsule; LV, lateral ventricle; Mesen, mesencephalon; Pu, putamen.

The inferior longitudinal fasciculus, the uncinate fasciculus and the inferior fronto-occipital fasciculus are ventral bundles respectively connecting the temporal lobe to the occipital lobe, insula to the frontal lobe, and the frontal lobe to the occipital lobe (Figure 2). The inferior fronto-occipital fasciculus is parallel and medial to the inferior longitudinal fasciculus within the occipital lobe and parallel to the uncinate fasciculus within the temporal lobe.

**Figure 3:**
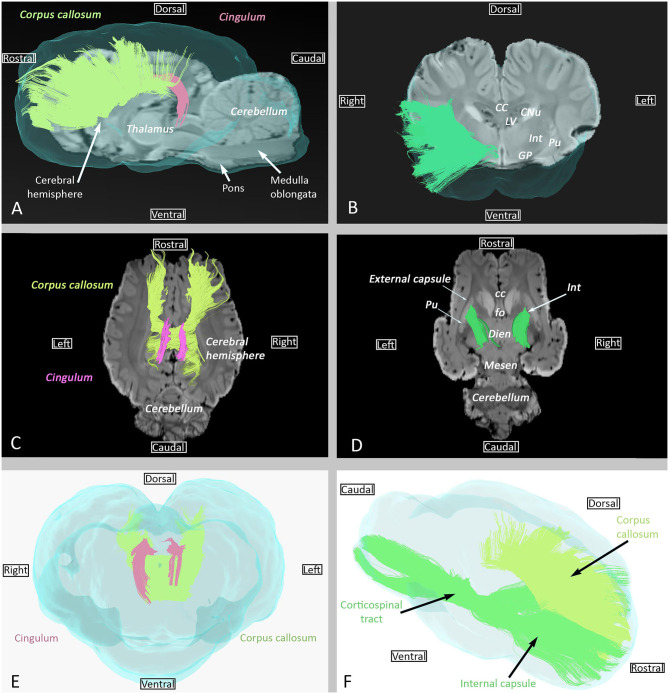
Examples of association (cingulum), commissural (corpus callosum), and projection (internal capsule) fibers. **(A)** Sagittal view of cingulum (pink) and corpus callosum (lime green) overlaid on SWI and the isosurface of the brain. **(B)** Transversal view of the internal capsule overlaid on SWI and the isosurface of the brain. **(C)** Dorsal view of cingulum (pink) and corpus callosum (lime green) overlaid on SWI. **(D)** Dorsal view of the internal capsule overlaid on SWI. **(E)** Transverse rostral view of cingulum (pink) and corpus callosum (lime green) overlaid on the isosurface of the brain. **(F)** Sagittal view of the internal capsule (green) and corpus callosum (lime green) overlaid on the isosurface of the brain. cc, corpus callosum; CNu, caudate nucleus; Dien, diencephalon; fo, fornix; GP, globus pallidus; Int, internal capsule; LV, Lateral ventricle; Mesen, mesencephalon; Pu, putamen.

The cingulum, which is part of the limbic system, is a medial bundle that runs in the depth of the cingulated gyrus. It is connected to the frontal, parietal, and occipital lobes (Figures 3A,C,E).

### Commissural Fibers

**Figure 4:**
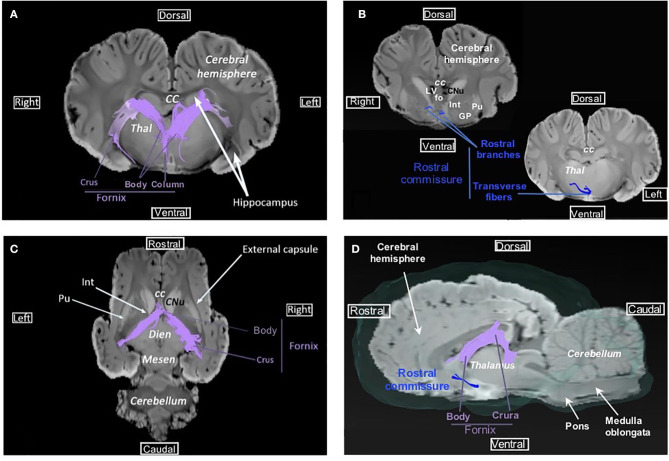
Fornix and rostral commissure. **(A)** Transversal view of fornix overlaid on SWI. **(B)** Transversal view of the rostral commissure (at the lentiform nucleus level and at the thalamus level) overlaid on SWI. **(C)** Dorsal view of fornix overlaid on SWI. **(D)** Sagittal view of the rostral commissure and fornix overlaid on SWI and the isosurface of the brain. cc, corpus callosum; CNu, caudate nucleus; Dien, diencephalon; fo, fornix; GP, globus pallidus; Int, internal capsule; LV, lateral ventricle; Mesen, mesencephalon; Pu, putamen; Thal, thalamus.

Commissural fibers, which cross the midline to connect homolog cortical areas, are present in three particular bundles: corpus callosum, rostral commissure, and fornix commissure (Figures 3, 4). The corpus callosum is composed of decussating fibers which connect neopallial areas. These fibers form the ceiling of the lateral ventricles and cross those of the corona radiata in the centrum semiovale (Figure 3F). The corpus callosum is divided into a rostral part (genu) connecting frontal areas, a central part (body) connecting parietal and temporal areas, and a caudal part (splenium) connecting occipital lobes. The rostral commissure is composed of transverse fibers located in the lamina terminalis, rostrally to the columns of the fornix (Figure 4D). The rostral and caudal parts of the rostral commissure are respectively composed of fibers running in the olfactory bulbs and tubercles and fibers crossing the rostral edge of the lentiform nucleus to reach the piriform and temporal lobes (Figures 4B,D). Fibers of the fornix commissure connect the two hippocampi. The fornix is made of two C-shaped bundles, one on each side, originating in the fimbria and running to the hypothalamus (Figures 4C, D). Joined to each other on the midline to form the body, these bundles are separated rostrally and caudally to form the columns and crura of the fornix, respectively.

### Projection Fibers

Projection fibers connect the cerebral cortex with other parts of the nervous system (deep nuclei, brainstem, cerebellum, and spinal cord). The internal capsule and corona radiata, the most important projection fiber complex, contain corticopetal fibers and corticofugal fibers. Corticopetal fibers connect the thalamus and metathalamus to the cerebral cortex; corticofugal fibers leave the cortex to reach the mesencephalon (corticomesencephalic tract), pons (corticopontine tract), cerebellum (cortico-ponto-cerebellar tract), ventral rhombencephalon (corticobulbar tract), and spinal cord (corticospinal tract). In this study, it was not possible to differentiate between these different tracts. The internal capsule is displayed in Figures 3B,D,F).

## Discussion

The goal of this study was to develop the methodology (imaging protocol and placement of seed ROI) for DTI tractography of the main white matter tracts of the horse. The three fiber groups (association, commissural, and projection fibers) known from classical anatomy have been successfully reconstructed by tractography using a formalin-fixed equine brain. A previous study conducted in the laboratory with a different reconstruction method had already shown the feasibility of tractography in an *ex vivo* equine brain (37) but had failed to identify the association pathways displayed in the present study. So this study is the first one, to our knowledge, to provide a complete anatomical description of the reconstructed fiber bundles of the equine brain.

Anatomy of the association, commissural, and projection fibers reconstructed in this study correlates well with the description of these fibers in anatomical textbooks. However, comparison of our results with those of the fiber dissection study of Pascalau et al. performed on equine brains shows discrepancies concerning the cingulum, for which the parietal and occipital radiations described by Pascalau et al. could not be observed in our study and the inferior fronto-occipital fasciculus was reconstructed in our study while it could not be identified by dissection (35). As DTI tractography provides an indirect characterization of axonal pathways based on the diffusion of water molecules, it hardly differentiates crossing and branching patterns in an individual voxel. Tractography algorithms can lead to false connections or premature terminations of tracked fibers and thus be responsible for an anatomical inaccuracy between MRI-derived reconstruction and dissection (43). Similarly to our findings regarding the inferior fronto-occipital fasciculus, reconstruction of tracts that could not be identified on gross anatomical dissection has already been reported for the feline inferior fronto-occipital fasciculus (24, 25, 27, 35) and the human superior fronto-occipital fasciculus (44, 45). For this latter fasciculus, modeling errors are assumed to have generated false continuations between different projection fibers, thus leading to the reconstruction of a continuous fronto-occipital fiber bundle (45). Even if modeling errors should be considered in our study, the trajectory of our reconstructed inferior fronto-occipital fasciculus (fibers coursing through the ventral external capsule and the stratum sagittale, Figures 2C,D) and the location relative to the uncinate and inferior longitudinal fasciculi (Figure 2F) are consistent with the literature (19, 20). Additional studies comparing tractography and dissection with a larger number of brains are needed to determine whether this fronto-occipital fasciculus is present or not in horses.

Other factors may have influenced the anatomical accuracy of tracked fibers, notably the operator dependence of ROI placement and the quality of *ex vivo* DTI data. Even if manual delineation of ROI is prone to generate biases, it was not possible to use automated segmentation as stereotaxic white matter atlases based on DTI are not available in the equine species. A freely available standard stereotaxic brain atlas was recently published for horses (16), but it does not contain DTI data. The creation of a tractography atlas could be a potential evolution of the present work. Indeed, once the imaging protocol and the methodology developed in this study are applied to a large number of horses, the reconstructed tracts could be co-registered to the equine stereotaxic brain atlas.

As the specimen used in this study is a brain extracted from the skull and fixed with formalin, the quality of MRI data may have been impacted both by fixation-induced tissue changes and by magnetic susceptibility artifacts at the tissue–air interface. For *postmortem* DTI acquisitions, the brain must be fixed in order to stop autolysis that degrades *in vivo* structural characteristics. While the fixation process causes a decrease in overall water diffusion compared to *in vivo*, its impact on diffusion anisotropy is still not well characterized with studies reporting a decrease in anisotropy in the fixed brain (46, 47) and others suggesting a preservation of relevant tissue microstructures by the fixative (48–51). In order to minimize the fixation-induced diffusion changes and their impact on the fiber tracking results, the brain was fixed within a very short time after death to avoid autolysis (52) and immersed in formalin for 1 month to allow homogeneous fixation of the whole brain (53), and the *b*-value was increased (3,000 s/mm^2^) in order to accommodate for the reduced diffusion of water molecules (46). Concerning magnetic susceptibility artifacts, the brain was immersed in a container filled with saline solution during acquisition in order to minimize them. Saline solution is meant to have an isosignal with the cerebrospinal fluid. Despite these precautions, *postmortem* conditions might have led to a decrease in the number of reconstructed fibers and/or to shorter fibers compared to what would have been obtained *in vivo* (47). The impact of fixation on the fiber outcome remains however difficult to estimate, since the few studies comparing *in vivo* and *ex vivo* tractography show variable results and quality of *ex vivo* tractography is also modulated by the fixation protocol, spatial resolution of images, diffusion weighting, and method of reconstruction (47, 54).

The use of advanced MRI techniques in clinical practice offers the opportunity to better understand the mechanisms of neurological diseases of the horse and to improve their diagnosis. In the research setting, the implementation of such advanced MRI techniques often use *postmortem* samples, as exemplified by the two most recent MRI studies of the normal equine brain (15, 16). Indeed, in addition to the constraints related to the adaptation of facilities for equine neuroimaging, the development of MRI tools in neurologically healthy horses poses the ethical problem of performing a general anesthesia for an MRI examination which is not medically warranted. Furthermore, using *postmortem* brains has the advantage of performing multiple imaging protocols in a single setting for comparison, as the scanning time is not a limitation. As access of horses to high-field MRI is likely to become more common in the future, the advanced MRI methods developed in *postmortem* samples may be soon translated into the clinical field. Hence, the DTI protocol of this study was implemented in order to achieve sufficient image quality for fiber tracking while maintaining an adequate scanning time for future *in vivo* use. This scanning time will be compatible with anesthesia as it may be significantly reduced in *in vivo* conditions. Indeed, repetition of acquisitions, which had to be carried out five times (12 min × 5) on average to obtain a greater signal in our *postmortem* study, will no longer be necessary as the signal will naturally be greater. Likewise, the *b*-value, which was high in this study (3,000) to be sensitive to the diffusion phenomenon, could be decreased to 1,000 in *in vivo* conditions for DTI deterministic tractography, possibly reducing the acquisition time.

Other methods of reconstruction could have been considered. DTI reconstruction was our first choice, since it is the most popular and widely used (1) for fiber exploration and thus the most reproducible. However, when a high angular resolution and thus a high number of directions are used for acquisition, other reconstructions can be performed. One of them is the Q-ball imaging (QBI) reconstruction which differs from DTI reconstruction by taking multiple fiber orientations into account instead of only giving an ellipsoid. Hence, QBI could be especially useful in the case of multiple fibers crossing in one voxel mentioned above. We plan to compare, in future studies, fiber tracking obtained with QBI to that obtained with DTI. Even if directions and *b*-value were initially chosen to be optimal for the DTI method, we ensured that our protocol was as close as possible to the future QBI acquisition.

The use of a single brain is an important limitation of our study, since it prevents the analysis of fiber bundle anatomy according to age, sex, and breed. Our study only provides the methodological tools (imaging protocol and placement of seed ROI) necessary for carrying out such anatomical studies. The use of a single brain also precludes any assessment of tract consistency from one brain to another. Since the anatomy of the reconstructed fiber bundles is consistent with the literature, the placement of ROIs seems to be appropriate and should normally lead to the same fiber tracking results in other brains. However, further large-cohort DTI studies are critical to validate and/or optimize the methodology developed in the present work and to investigate the characteristics of the equine white matter anatomy.

To conclude, our results demonstrate the feasibility of using *postmortem* DTI data to reconstruct the main white matter tracts of the equine brain. Further DTI acquisitions and corresponding dissections of equine brains are needed to validate these findings and create a tractography atlas that could be used in equine neuroimaging research.

## Data Availability Statement

The datasets generated for this study are available on request to the corresponding author.

## Ethics Statement

This study used animal tissue after euthanasia and was exempt from ethics approval. Written informed consent was obtained from the owners for the participation of their animals in this study.

## Author Contributions

SB: writing—original draft, conceptualization, methodology, and visualization. GA: writing—original draft, conceptualization, and methodology. BC: conceptualization and resources. EL: resources. PP: supervision. AD and GM: writing—review and editing and conceptualization. All authors contributed to the article and approved the submitted version.

## Conflict of Interest

The authors declare that the research was conducted in the absence of any commercial or financial relationships that could be construed as a potential conflict of interest.
