# Supplementary material for: Diffusion Tensor Imaging Tractography of White Matter Tracts in the Equine Brain
Source: Front Vet Sci. 2020 Jul 30;7:382. doi: 10.3389/fvets.2020.00382 (PMC7406683; doi:10.3389/fvets.2020.00382)
Supplement: Supplementary file 1 [file Data_Sheet_1.docx]

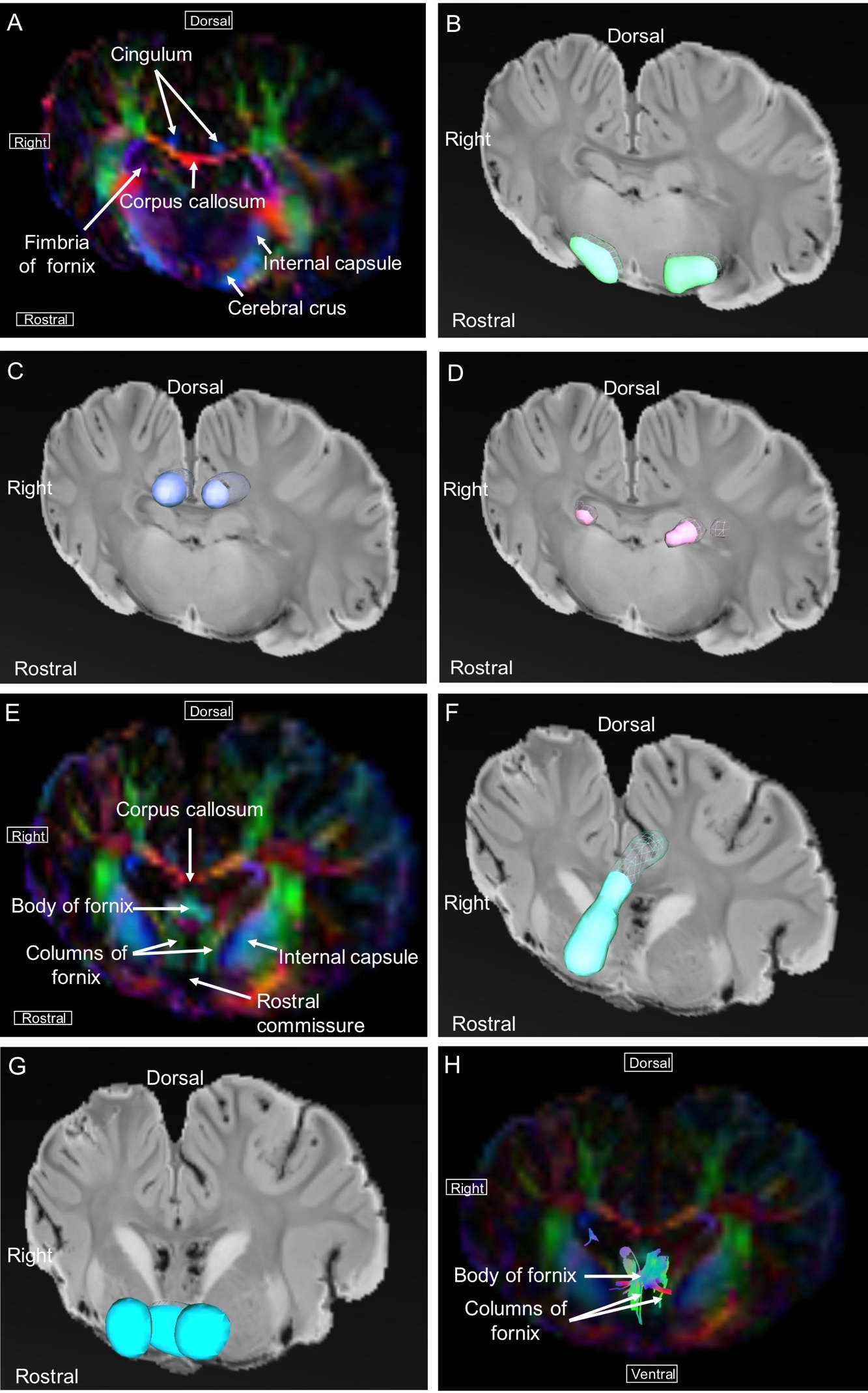


**Supplementary Figure 1 : Delineation of ROI on transverse slices of the equine brain : FA-weighted color maps (A,E,H) and three-dimensional representation of ROI overlaid on susceptibility-weighted images (B,C,D,F,G).**

On color-coded FA maps, the color represents the orientation of tissue alignment: green : ventral to dorsal (or dorsal to ventral), blue : rostral to caudal (or caudal to rostral); red : right to left (or left to right). On susceptibility-weighted images, the solid part of the ROI corresponds to the volume located rostrally to the section plane presented; the dashed part of the ROI corresponds to the volume located caudally to the section plane presented.

**(A-D) Transverse slices of the equine brain at the level of the cerebral crura.**

The internal capsule (blue fibers), cerebral crura (blue fibers), cingulum (blue fibers), corpus callosum (red fibers) and fimbriae of the fornix (purple) are all resolvable on color-coded FA maps (A). A two ROI approach is used to reconstruct the internal capsule/corona radiata : the first one, presented on the transverse susceptibility-weighted image, corresponds to the cerebral crura, the second one (*not shown*) is defined as the brainstem (B). A single ROI (cingulum) and a ROA (corpus callosum) are delineated on about ten slices for the reconstruction of the cingulum (C). The fimbriae of the fornix are delineated on about ten slices for the reconstruction of the fornix (D).

**(E-H) Transverse slices of the equine brain at the level of the rostral commissure.**

Commissural fibers (body and columns of fornix, corpus callosum and the transverse fibers of the rostral commissure) are all identifiable on FA-weighted color maps (E). The corpus callosum is delineated from the rostrum to the splenium (F). For the reconstruction of the rostral commissure, ROI are defined around the rostral branches and the transverse fibers of this bundle (G). For the corpus callosum and the rostral commissure, once the ROI are delineated on transverse planes, the accuracy of their placement is checked on sagittal slices (*not shown*). As the fornix could be reconstructed with the sole delineation of the fimbriae, the body of the fornix was not included in the definition of the ROI (H).


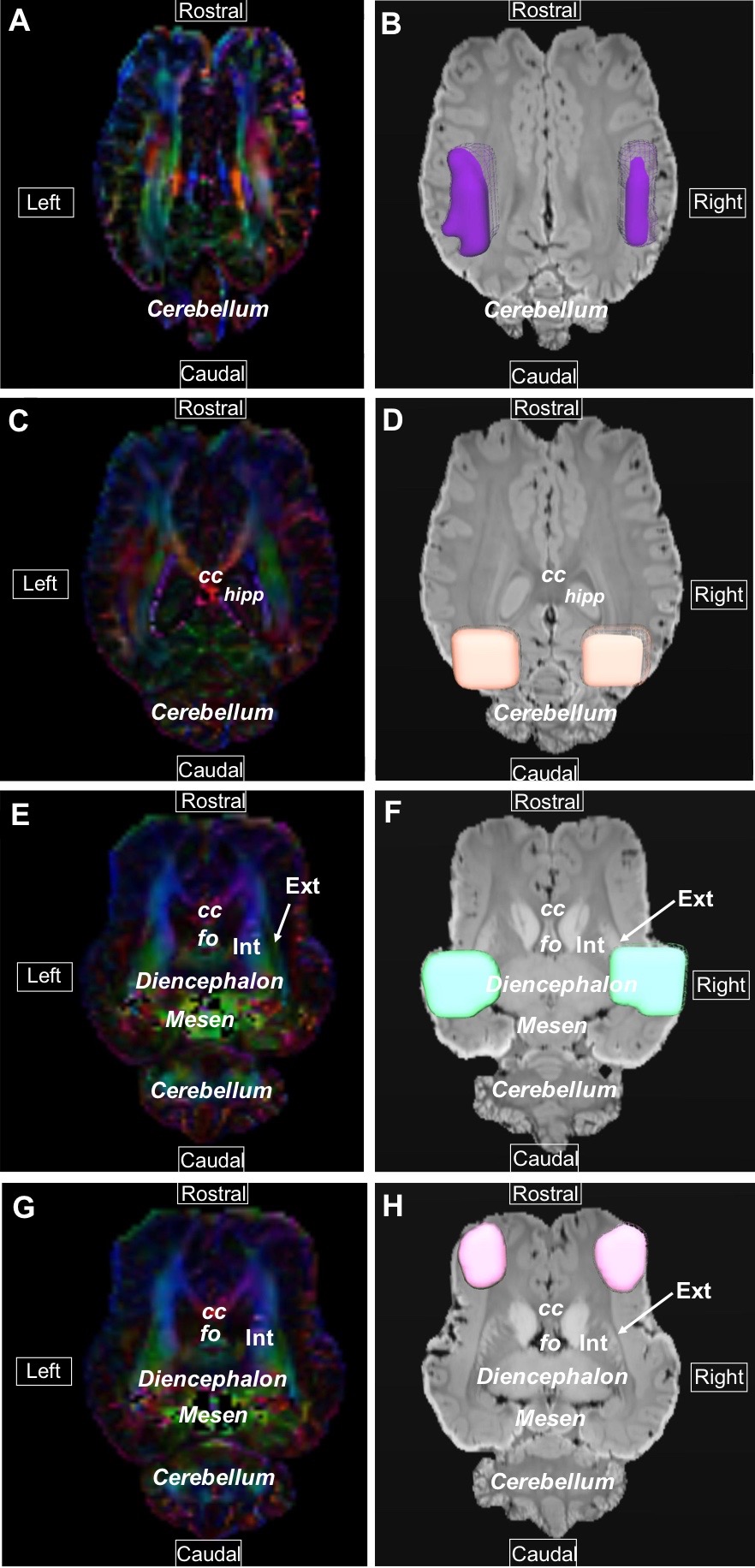


**Supplementary Figure 2 :** **Delineation of ROI on dorsal slices of the equine brain: FA-weighted color maps (A-C-E-G) and three-dimensional representation of ROI overlaid on susceptibility-weighted images (B-D-F-H).**

On color-coded FA maps, the color represents the orientation of tissue alignment: green : ventral to dorsal (or dorsal to ventral), blue : rostral to caudal (or caudal to rostral); red : right to left (or left to right). On susceptibility-weighted images, the solid part of the ROI corresponds to the volume located dorsally to the section plane presented, the dashed part of the ROI corresponds to the volume located ventrally to the section plane presented.

(A-B) Delineation of the ROI « Arcuate fasciculus »

The white matter underneath the bottom part of the frontal, parietal and temporal sulci is delineated from the dorsal to the ventral region. The most dorsal slice is above the lateral ventricles and the corpus callosum and the most ventral slice is defined as the first slice where the internal capsule can be identified.

(C-D) Delineation of the ROI “occipital”

The white matter of the occipital lobe is delineated from the dorsal to the ventral region: from the slice above the lateral ventricles and the corpus callosum to the first slice where the temporal horns of the lateral ventricles can be identified. This ROI is used for the reconstruction of the inferior longitudinal fasciculus (along with the ROI “temporal”) and the inferior fronto-occipital fasciculus (along with the ROI “frontal”).

(E-F) Delineation of the ROI “temporal”

The white matter of the temporal lobe is delineated from the dorsal to the ventral region: from the first slice where the temporal horns of the lateral ventricles can be identified to the last slice exhibiting temporal lobe. This ROI is used for the reconstruction of the inferior longitudinal fasciculus (along with the ROI “occipital”) and the uncinate fasciculus (along with the ROI “frontal”).

(G-H) Delineation of the ROI “frontal”

The white matter of the frontal lobe is delineated from the dorsal to the ventral region on about five slices, starting from the first slice where the temporal horns of the lateral ventricles can be identified. This ROI is used for the reconstruction of the inferior fronto-occipital fasciculus (along with the ROI “occipital”) and the uncinate fasciculus (along with the ROI “temporal”).

cc : corpus callosum, Ext : external capsule, fo: fornix, hipp: hippocampus, Int: internal capsule, Mesen : mesencephalon.
